# GLP-1 Agonists and the Risk of Pulmonary Aspiration during Elective Upper Endoscopy: A Systematic Review and Meta-analysis

**DOI:** 10.2174/0118743064372550250603061720

**Published:** 2025-06-11

**Authors:** Praveen Reddy Elmati, Gowthami Sai Kogilathota Jagirdhar, Rakhtan K. Qasba, Andres Perez, Ruman K. Qasba, Yatinder Bains, Mehul Shah, Salim Surani

**Affiliations:** 1 Department of Anesthesiology, Saint Clare’s Hospital, Dover, NJ 07081, USA; 2 Department of Gastroenterology, Saint Michaels Medical Center, Newark, NJ 07104, USA; 3 Department of Medicine, Green Life Medical College and Hospital, Dhaka, Bangladesh; 4 Department of Medicine, Saint Francis Health Systems, Tulsa, NJ, USA; 5 Department of Medicine, Sher-i-Kashmir institute of medical sciences, Srinagar, Jammu and Kashmir, India; 6 Department of Medicine & Pharmacology, Texas A&M University, College Station, TX 77843, Texas, USA

**Keywords:** Endoscopy, Pulmonary aspiration, GLP-1 receptor agonist, Periprocedural complications, Aspiration complication, Systematic Review, Elective endoscopy

## Abstract

**Introduction:**

Glucagon-like Peptide-1 (GLP-1) agonists cause delayed gastric emptying by acting on vagal afferent nerves. Retained gastric contents (RGC) increase the risk of pulmonary aspiration, particularly under anesthesia in endoscopic procedures. This systematic review and meta-analysis aim to summarize the current evidence on pulmonary aspiration in patients receiving GLP-1 agonists undergoing endoscopy.

**Methods:**

A systematic review was conducted using Cochrane, Embase, and PubMed from inception to May 2024, including studies and case reports examining GLP-1 agonists and pulmonary aspiration. Data on study characteristics, patient demographics, and GLP-1 agonist use were collected. A pooled analysis of retrospective studies was performed using RevMan version 5.4.1. The study protocol was registered in the PROSPERO database (ID CRD42024595241).

**Results:**

A total of five case reports involving six patients and twelve studies including 210,216 patients were identified. Pulmonary aspiration occurred in 143 of 87,691 patients (0.16%) in the GLP-1 agonist group and 149 of 122,525 patients (0.12%) in the placebo group. Notably, three patients experienced aspiration despite stopping GLP-1 agonists more than six days prior and fasting for over eight hours. The meta-analysis showed an odds ratio of 1.23 (P = 0.59; 95% CI, 0.58 to 2.60) for pulmonary aspiration associated with GLP-1 agonist use, which was not statistically significant.

**Discussion:**

This analysis did not find a statistically significant association between GLP-1 agonist use and pulmonary aspiration risk during endoscopic procedures. While the findings align with some existing studies suggesting minimal increased risk, the presence of aspiration cases despite prolonged fasting highlights potential gaps in current peri-procedural management. Limitations include reliance on retrospective data and case reports, as well as variability in fasting protocols.

**Conclusion:**

The study found no significant association between GLP-1 agonist use and pulmonary aspiration risk during endoscopy. Further research is warranted to develop evidence-based fasting guidelines and optimize peri-procedural management for patients on GLP-1 agonists.

## 1 INTRODUCTION

Glucagon-like Peptide-1 (GLP-1) receptor agonists, such as semaglutide, and synthetic dual agonists targeting both glucose-dependent insulinotropic polypeptide and GLP-1 receptors, such as tirzepatide, have been gaining popularity due to their weight loss properties and related benefits. Due to their potential as an alternative to bariatric surgery, patients frequently request prescriptions from their healthcare providers [1, 2]. However, the prevalence of nausea and other gastrointestinal symptoms is often the primary factor limiting continued use [3, 4]. Despite this, evidence in the literature suggests that these side effects are typically temporary and tend to resolve with continuation.

Glucagon-like peptide-1 (GLP-1) agonists work by enhancing insulin secretion and inhibiting glucagon release. Locally in the gastrointestinal tract, they delay gastric emptying by reducing antral and duodenal motility while increasing pyloric tightening. During anesthesia, delayed gastric emptying is a known risk factor for pulmonary aspiration [5]. Semaglutide, administered as a once-weekly injection, has gained significant attention from both medical practitioners and patients due to its favorable safety profile and low incidence of gastrointestinal side effects [1].

While the effects of GLP-1 agonists have been extensively studied in the context of weight loss and glucose regulation, their impact on delayed gastric emptying and the associated risks of Retained Gastric Contents (RGC) and pulmonary aspiration, remains less understood. For patients on GLP-1 agonists who require elective, urgent, or emergent procedures, optimal preoperative management is still under debate. Currently, there are no evidence-based guidelines, particularly regarding endoscopic procedures, on whether to hold these medications or how to adjust fasting protocols for this patient population.

Fujino *et al*. described a case of delayed gastric emptying in an obese 31-year-old female patient taking semaglutide for type 2 diabetes mellitus, even after following the American Society of Anesthesiologists recommendations for fasting guidelines. On endoscopy, food residue was found in the gastric body, and the procedure had to be aborted [6]. Strategies to decrease the risk of aspiration, like holding the medication up to four weeks before a scheduled procedure, were proposed by Gulak *et al*. after a case of a nondiabetic, nonobese patient with unexpected regurgitation of a large volume gastric content, despite a 20-hour solid and an 8-hour liquid fasting liquid [7]. Sherwin *et al.*, using point-of-care (POC) ultrasound, showed that GLP-1 alters gastric emptying and retained gastric contents (RGC) two hours after clear liquid intake and after an overnight fast for solid food. This is concerning for patients who typically fast overnight before a procedure requiring anesthesia [8]. In a single-center electronic chart review, Silveira *et al.* found that patients taking semaglutide had increased residual gastric contents during elective esophagogastroduodenoscopy. However, interruption of preoperative semaglutide for 10–14 days was not predictive of increased RGC (24% vs. 5%) [9].

However, the American Gastroenterological Association, American Association for the Study of Liver Diseases, American College of Gastroenterology, American Society for Gastrointestinal Endoscopy, and North American Society for Pediatric Gastroenterology, Hepatology, and Nutrition released a joint statement on August 11, 2023, noting a lack of concrete evidence on best practices for patients on GLP-1 agonists and insufficient data on whether to stop medication before endoscopy [10]. They encouraged further studies focusing on these endpoints. The American Society of Anesthesiologists has provided consensus-based guidance suggesting holding GLP-1 agonists for one week in patients on weekly dosing and for one day in those on daily dosing prior to endoscopic or surgical procedures to reduce aspiration risk [11]. We, therefore, conducted a meta-analysis on pulmonary aspiration in patients on GLP-1 agonists undergoing endoscopy to guide perioperative management and fasting recommendations.

## 2 METHODS AND MATERIALS

This review is reported following the PRISMA (Preferred Reporting Items for Systematic Reviews and Meta-Analyses) guidelines, as indicated in the PRISMA checklist [12]. This systematic review, along with meta-analysis, is registered in the PROSPERO international database (ID CRD42024595241; www.crd.york.ac.uk /prospero).

### 2.1 Data Sources, Study Search, and Inclusion and Exclusion Criteria

A thorough literature search was performed using three bibliographic databases, Cochrane, Embase, and PubMed, from inception to May 2024. Using a combination of keywords and medical subject headings (MESH), we used vocabulary related to “GLP-1 agonists” OR “GLP-1 analog” OR “liraglutide” OR “semaglutide” OR “exenatide” OR “lixisenatide” OR “albiglutide” OR “dulaglutide” OR “tirzepatide” AND “pulmonary aspiration” OR “aspiration”.

Four authors (GSKJ, AP, RKQ, and RKQ) participated in the process of study selection. After duplicates were removed using Endnote reference manager software, two authors independently screened the titles and abstracts. This process was conducted using the Rayyan software (https://rayyan.ai/). Studies meeting the inclusion criteria were retrieved and assessed for full-text eligibility. Disagreements between the two authors regarding study selection were resolved through discussion, or by involving a third arbitrator if consensus could not be achieved.

We included studies involving adult patients aged 18 years or older using GLP-1 agonists. All healthy, obese, and diabetic patients were eligible. The control population consisted of patients on placebo or not using GLP-1 agonists. Studies assessing aspiration, nausea, vomiting, Gastric Emptying Rate (GER), and fasting gastric content, volume, or residue were also included. We excluded studies that were (i) conducted on animals, (ii) unpublished, or (iii) written in languages other than English. Additionally, we reviewed references in published manuscripts on the topic to identify relevant studies that met the criteria but were not retrieved during the initial literature search.

### 2.2 Data Extraction

Three authors (AP, RKQ, and RKQ) independently extracted information, including general details (authors, DOI, title, journal, publication year), study and participant characteristics (study design, location, study duration, sample sizes in the GLP-1 and control groups, type of GLP-1 agonist used), and outcomes related to pulmonary aspiration. All the data were transferred into a pre-piloted extraction form in Google Sheets. A fourth author (Jagirdhar GSKJ) checked the extracted data independently for validity.

### 2.3 Analysis of Results

from the extracted data. Continuous variables were reported as median (IQR) or mean (± SD), categorical data as percentages, and outcomes as both numbers and percentages. A computer application, Review Manager (RevMan, version 5.4.1; Cochrane Collaboration, 2020), was used to analyze all results [13]. For each study outcome, odds ratios (ORs) and 95% confidence intervals (CIs) were calculated using a random-effects model [14] based on the number of events and non-events. A p-value of <0.05 was considered statistically significant. Meta-analysis was performed, and forest plots were generated. Cochrane’s Q test and the I^2^ statistic were used to assess heterogeneity across studies [14], with low heterogeneity defined as I^2^ = 20% [14]. Funnel plots were used to evaluate the likelihood of publication bias, and sensitivity analysis was conducted to assess the robustness of the results [15].

### 2.4 Quality Assessment

To check the quality of the studies, the National Institutes of Health scale was used to evaluate case-control and cohort studies (https://www.nhlbi.nih.gov/ health-topics/study-quality-assessment-tools). Based on the scale, studies were classified as good, fair, or poor. Case reports were assessed through the Joanna Briggs Institute (JBI) critical appraisal checklist (https://joannabriggs.org/) [16]. Two authors (Qasba RK, Qasba RK) independently performed the quality appraisal of the included studies, and any disagreements were resolved through discussion or a third arbitrator (Kogilathota JagirdharGS).

## 3 RESULTS

### 3.1 Search and Selection

**Fig. (1):**
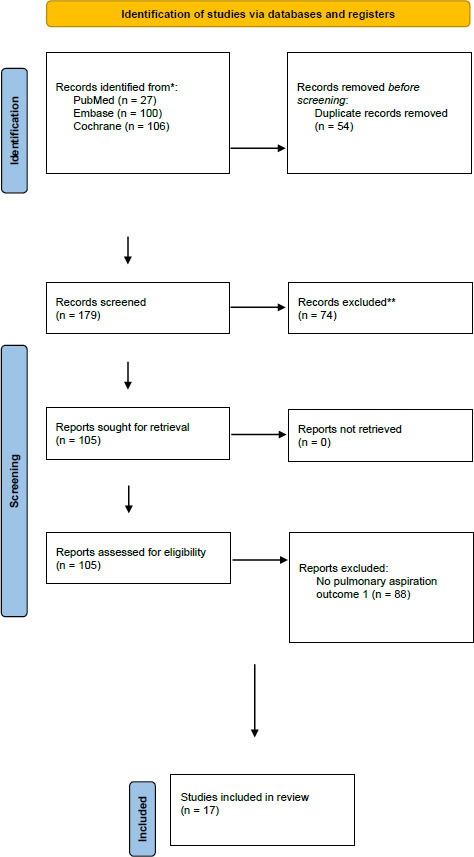
Prisma flowchart for the process of study selection and inclusion.

A total of 233 records were identified on the initial search, out of which fifty-four were identified as duplicates; after completion of title and abstract screening, 105 were chosen for full-text screening. Seventeen studies were deemed to meet the inclusion criteria. Out of the final seventeen selected studies, five were identified as case reports and twelve as retrospective studies. (Fig. **1**) demonstrates the PRISMA diagram underlying the study selection.

### 3.2 Characteristics of the Included Studies Case Reports

Our analysis included five case reports with six patients [5-7, 17, 18]. In the included case reports, semaglutide was used as a GLP-1 agonist, with doses ranging from 0.25, 0.5, and 1.7 mg once a week. The indication for the GLP-1 agonist was obesity. The duration of fasting before endoscopy ranged from 8 hours to 20 hours, and medication was stopped for >= 6 days before the procedure. Table **1** shows the characteristics of the included case reports on GLP-1 agonists in patients undergoing endoscopy.

### 3.3 Retrospective Studies

The analysis included twelve studies [9, 19-29] with a total number of 210, 216 patients, out of which 87691 (0.16%) used GLP-1 agonists and 122525 (0.12%) were on placebo. Most of the studies were conducted in the USA except one in the Netherlands [28]. In the majority of studies, GLP-1 agonists were indicated for obesity or Type 2 DM. Four studies did not mention the type of sedation used [20, 22, 24, 27]. Zaffar *et al.* included patients under deep sedation/general anesthesia [28], and Wu *et al.* used EGD under anesthesia but did not specify the type of sedation used [30]. Silveira *et al.* used deep sedation or general anesthesia [9]. Garza *et al.* stated that all procedures were performed with monitored anesthesia care [23]. Further, none of the studies analyzed results based on the type of anesthesia used. Barlowe *et al.*, Nadeem, Kumar, and Yeo *et al.* used ICD9 and ICD10 codes to define aspiration [20, 22, 24, 27]. However, Garza, Silveira, Zaffar, and Wu *et al.* did not mention the definition of pulmonary aspiration used in their studies [9, 19, 23, 28]. Moreover, POC ultrasound was not used prior to endoscopy to determine RGC in any of the case reports or studies included in our manuscript.

Out of 210,216 patients, 143/87691 (0.16%) in the GLP-1 agonist and 149/122525 (0.12%) in the placebo group had pulmonary aspiration. The meta-analysis showed that GLP-1 agonists were associated with 1.23 odds of pulmonary aspiration (P= 0.59 and 95% CI of 0.58 to 2.60, not statistically significant). Fig. (**2**) shows the Forest plot and meta-analysis for Pulmonary aspiration in patients on GLP-1 agonists and placebo, and Fig. (**3**) shows the sensitivity analysis for pulmonary aspiration in patients on GLP-1 agonists and placebo. The study by Kumar *et al.* (2024) was excluded from the sensitivity analysis due to its large sample size and high statistical weight (29%), which disproportionately influenced the overall pooled effect. Although methodologically sound, the study accounted for the majority of aspiration events. Its exclusion significantly reduced heterogeneity in the

meta-analysis. Therefore, it was removed in the sensitivity analysis to assess the stability of the overall findings and evaluate potential outlier effects.

Supplementary Table (**S1**) shows the funnel plot for publication bias on pulmonary aspiration in GLP-1 agonists and placebo patients. The asymmetry in the plot suggests potential publication bias or heterogeneity in the included studies.

### 3.4 Quality Assessment

Most of the included case reports were assessed as being of good quality. The majority of studies clearly described the patients’ characteristics and clinical conditions. The current clinical condition was well-defined in all cases except in the study by Queiroz *et al.* [17]. Additionally, most reports provided a takeaway lesson, with the exception of Klein *et al.* [18]. Furthermore, all reports included clear descriptions of complications and the post-procedural clinical condition. Table (**S2**) shows the quality assessment of the included case reports.

Most of the included retrospective cohort studies were assessed as fair quality, with no studies rated as poor quality. Except for Zaffar *et al.* [28], all studies recruited subjects from similar populations and applied inclusion and exclusion criteria uniformly. Silveira *et al.*, Wu *et al.*, Yeo *et al.*, Barlowe *et al.*, and Maselli *et al.* [9, 20, 24, 25, 19] discussed the rationale for participant selection and provided justification for their sample sizes. However, most studies scored poorly in areas such as blinding of outcome assessors and repeated measurement of exposure. One case-control study was also assessed as fair quality. Tables (**S3** and **S4**) present the quality assessments of the included retrospective cohort and case-control studies, respectively.

## 4 DISCUSSION

Our systematic review and meta-analysis of seven retrospective studies and five case reports aimed to evaluate the adverse effect of GLP-1 agonists on pulmonary aspiration events during endoscopic procedures. The odds of aspiration in patients on GLP-1 receptor agonists were 1.45. However, the results were not statistically significant (P = 0.47). The case reports predominantly involved semaglutide, suggesting it may be a significant contributor to pulmonary aspiration among patients on GLP-1 agonists. In these reports, aspiration occurred despite withholding semaglutide for more than 6 days and across a range of doses, indicating that neither dose nor withholding duration alone reliably mitigated the risk. If gastric emptying from semaglutide, a long-acting agent, achieves tachyphylaxis over time, pulmonary aspiration may be due to factors other than GLP-1 agonist use.

In our manuscript, the majority of observational studies used long-acting GLP-1 agonists. There was no difference in pulmonary aspiration based on long-acting GLP-1 agonist use.

Compared to our meta-analysis, which included a broader population and various GLP-1 agonists, the case reports may have introduced a bias toward semaglutide-specific effects. However, since case reports typically highlight severe or unusual outcomes, their inclusion might have exaggerated the perceived risk and may not reflect the true incidence in larger populations. This is reflected in our pooled meta-analysis estimate, which shows no statistically significant increase in aspiration risk (OR 1.23; 95% CI 0.58–2.60) and substantial heterogeneity (I^2^ = 75%).

Therefore, while the case reports provide important clinical context, they should be interpreted with caution given their inherent limitations, including lack of control groups, publication bias, and selective reporting.

A high level of heterogeneity was observed in our meta-analysis. This may be attributed to differences in the type of GLP-1 agonist used, duration of use, the length of time the medication was held prior to the procedure, and fasting periods. Additionally, the depth of sedation—including monitored anesthesia care, general anesthesia, and deep sedation—varied across studies, as did the methods used to identify aspiration events. Since pulmonary aspiration risk is strongly influenced by baseline patient characteristics, factors such as indications for GLP-1 agonist use (e.g., diabetes and obesity) and underlying comorbidities like GERD and autonomic neuropathy may also contribute to the observed heterogeneity.

Pulmonary aspiration is a known complication of gastrointestinal endoscopy, occurring at a rate of 4.6 per 10,000 endoscopies in a retrospective study by Bohman *et al.* Upper airway manipulation and stomach insufflation during the procedure can increase regurgitation and the risk of aspiration [30]. In our meta-analysis, the placebo group had nearly three times the aspiration rate compared to Bohman *et al.* and similar studies. This elevated risk in the placebo group may be attributed to several patient-related factors that differ significantly from the general endoscopy population in prior research. Conditions, such as diabetes, obesity, sedation-related suppression of airway reflexes, and comorbidities like autonomic neuropathy could contribute to this increased risk. The placebo group may represent a subset of patients with higher metabolic and gastrointestinal risk profiles, rather than reflecting the broader, generally healthier population undergoing routine endoscopy, as studied by Bohman *et al.*

Interest in aspiration risk associated with GLP-1 agonists arose after observations that patients on these medications frequently had residual gastric contents despite adhering to standard fasting guidelines [31]. Stark *et al.* reported a fourfold increase in residual gastric content, with 4/49 patients on GLP-1 agonists compared to 2/188 in the control group [31]. Similarly, Kobori *et al.* found a tenfold increase in residual gastric content in their propensity-matched study, 5.4% versus 0.49% in controls (P = 0.004) [32].

However, studies examining residual gastric contents and aspiration events related to GLP-1 agonists remain small in size,with some lacking statistical significance [19, 31, 32]. Case reports have documented residual gastric contents despite fasting longer than usual in patients on GLP-1 receptor agonists [33]. There is speculation that the recent initiation of GLP-1 agonists may increase the incidence of delayed gastric emptying, while tachyphylaxis from vagal nerve activation reduces these effects with long-term use or higher doses [11, 34].

Short-acting GLP-1 agonists (exenatide, lixisenatide) primarily delay gastric emptying, leading to suppression of postprandial hyperglycemia. The side effects of gastric emptying are more pronounced with short-acting formulations than with long-acting ones (liraglutide, injectable semaglutide, weekly exenatide, lemaglutide, dulaglutide, albiglutide). In a study by Drucker *et al.* comparing twice-daily short-acting exenatide with weekly exenatide [35], short-acting exenatide maintained its delayed gastric emptying effect after 14 weeks of continuous administration, whereas long-acting exenatides showed evidence of tachyphylaxis over days to weeks. There is a lack of studies examining the relationship between short-acting GLP-1 agonists and pulmonary aspiration in the literature. It is also speculated that in patients with underlying gastroparesis, GLP-1 agonists may further reduce gastric emptying.

The American Society of Anesthesiologists, in their guidance released on June 29, 2023, recommends holding GLP-1 agonists for one week before the procedure in patients on weekly dosing, and one day before in those on daily dosing. However, these recommendations are based on expert opinion rather than strong evidence [11].

The ASA also recommends monitoring upper gastrointestinal symptoms and fasting for 8 hours on solids and 2 hours on liquids in patients without upper GI symptoms. However, this may be inadequate, as not all patients with RGC exhibit upper GI symptoms [11]. Similar to the joint statement from gastroenterology societies, there is insufficient concrete evidence linking GLP-1 agonist use to an increased risk of pulmonary aspiration. Therefore, based on our study results, we cannot recommend withholding GLP-1 agonists prior to procedures.

Recommendations for a 24-hour clear liquid diet to reduce aspiration events have been proposed based on studies by Ghazanfar *et al.* [29], Silveira *et al.* [9], Nasser *et al.* [36], and Maselli *et al.* [25]. However, additional evidence directly comparing pulmonary aspiration rates between a 24-hour clear liquid diet and the standard 8-hour fasting period, especially in patients with diabetes and obesity, is needed before this strategy can be widely implemented for those on GLP-1 agonists.

Standard fasting times help reduce the risk of aspiration during procedures. Some studies and case reports suggest using POC ultrasound to detect RGC in patients on GLP-1 agonists, especially those who are symptomatic. Aborting procedures in patients with RGC can help avoid complications by delaying the procedure or modifying anesthetic plans [8, 37]. Until evidence-based guidelines define the optimal duration for holding GLP-1 agonists before procedures, POC ultrasound may be a useful tool to assess aspiration risk. In a study of twenty patients, Sherwin *et al.* demonstrated that POC ultrasound detected RGC even after an 8-hour fast [8].

ImplementingPOC ultrasound in clinical practice requires training gastroenterologists or anesthesiologists to identify RGC. This presents several challenges, including the need for structured training, a thorough understanding of gastric physiology, and managing subjective interpretation. Additionally, trainees must be familiar with anatomical variations, such as gastric bypass, obesity, and other causes of altered anatomy. Other factors to consider include logistics, time constraints, equipment availability, and the development of standardized protocols for clinical decision-making.

The type of sedation used can also affect the risk of aspiration. Rezaiguia-Delclaux *et al.* state that propofol is associated with a higher risk due to impaired airway protection reflexes [38]. Intubation can protect the airway and decrease the risk of aspiration. However, endoscopies are usually performed under Monitored anesthesia care (MAC)/ deep sedation, leaving the airway unprotected and causing loss of protective airway reflexes leading to aspiration. This is evidenced in the retrospective study by Bohman *et al.*, where MAC sedations were associated with the highest rate of aspirations (6.9%) compared to general endotracheal intubation (6.4%) and registered nurse sedation (moderate intravenous (IV) sedation with fentanyl and midazolam) (0.9%).

## 5 IMPLICATIONS FOR CLINICAL PRACTICE

The risk of pulmonary aspiration in patients on GLP-1 agonists is still inconclusive. Pre-procedural patient assessment, symptomatic evaluation, and, if needed, POC ultrasound may help identify patients at risk of aspiration events. There is limited evidence in the literature on the use of pro-motility agents to decrease the risk of pulmonary aspiration in patients on GLP1 agonists undergoing medical procedures. Promotility agents like metoclopramide and erythromycin may be useful for emergency procedures based on POC ultrasound findings or patient's comorbidities for the high risk of retained gastric contents. Singh *et al*. concluded in their study that the management of GLP-1 in the perioperative period should be individualized and based on communication with anesthesiologists before endoscopies [39]. Anesthesiologists should be quick to recognize gastric contents during emergency procedures and take measures to avoid aspiration, including rapid sequence induction, head elevation, and intubation if necessary. Rescheduling elective procedures may also be considered.

## 6 STRENGTHS AND LIMITATIONS

We conducted a rigorous meta-analysis following established methodologies and adhering to the PRISMA guidelines. Additionally, we registered our study in PROSPERO to ensure transparency and reproducibility. We strictly followed the pre-defined study protocol throughout our meta-analysis. The majority of the evidence in our manuscript included case reports, retrospective case-control, and cohort studies, which are inherently associated with a risk of bias, particularly when confounding factors are not adequately adjusted [40, 41]. Due to the retrospective nature of these studies, it is important to note that our findings do not establish a causal relationship between GLP-1 agonists and pulmonary aspiration.

It is worth mentioning that all the case reports and retrospective studies focused on long-acting GLP-1 agonists, and there was a notable lack of evidence regarding short-acting GLP-1 agonists. Further, many of the studies did not have information on the dose, duration of usage, duration of holding GLP-1 agonists, and fasting period prior to the procedure. Depth of sedation has a significant effect on the risk of aspiration during endoscopy procedures. Multiple studies in our meta-analysis did not mention the depth of sedation and anesthesia used. Importantly, aspiration events were identified using ICD codes in some studies, while other studies did not clearly define the method of identifying aspiration, introducing variability in outcome assessment. These factors could have contributed to the high heterogeneity noted in our meta-analysis.

Although the funnel plot demonstrates some asymmetry, potentially suggesting publication bias, our exhaustive literature search did not identify any unpublished studies that met the inclusion criteria. The asymmetry observed on funnel plots may be attributable to methodological heterogeneity, including variation in sample sizes, population differences, and outcome definitions across the included studies. It is also possible that the skewed distribution reflects selective reporting and inherent differences in effect sizes among smaller studies. If unpublished studies with null or negative findings had existed and been included, the overall effect estimate might have been attenuated. These considerations highlight the importance of interpreting our findings in the context of the available published evidence.

Furthermore, the majority of the studies analyzed were conducted in the United States; however, we believe that our findings are generalizable to broader populations.

## IMPLICATION FOR RESEARCH

Due to conflicting evidence and the small sample sizes of current studies, further retrospective research is needed. Future studies should aim to clarify the relationship between GLP-1 agonist use and aspiration risk, including factors such as duration of use, type of GLP-1 agonist (short- vs. long-acting), and underlying comorbidities like diabetes and obesity. Key variables to investigate include fasting times, residual gastric contents, and aspiration events. Additionally, risk should be assessed for individual drugs, the time required to develop tachyphylaxis to delayed gastric emptying, and the optimal cessation period needed to normalize gastric emptying. The effective fasting duration prior to procedures in patients on GLP-1 agonists also warrants further investigation.

In patients at high risk for retained gastric contents, extending the fasting period by a few additional hours beyond current recommendations may not consistently reduce gastric volume. This warrants further investigation. The potential benefits of withholding solid food and adopting a 24-hour clear liquid diet—similar to preparation for colonoscopy, should be evaluated. Additionally, the efficacy of pro-motility agents, such as metoclopramide and erythromycin, in accelerating gastric emptying in these patients should be explored in future studies [42].

## CONCLUSION

This systematic review and meta-analysis found no statistically significant association between GLP-1 agonist use and increased risk of pulmonary aspiration during endoscopic procedures, though substantial heterogeneity was found among the included studies. Given the limitations in study design, inconsistent definitions of aspiration events, variation in GLP-1 agonist used and the absence of data on short-acting GLP-1 agonists, the evidence remains inconclusive.

Despite this, clinical vigilance is needed during endoscopy, particularly in patients with diabetes, obesity, or other risk factors for delayed gastric emptying. Until more robust evidence is available, we recommend individualized risk assessment for patients on GLP-1 agonists undergoing endoscopy. Point-of-care (POC) ultrasound may serve as a useful tool to identify retained gastric contents in high-risk or symptomatic patients, helping inform peri-procedural decision-making. Future studies are needed to determine optimal fasting times, the role of specific GLP-1 formulations, and the effectiveness of alternative strategies, such as clear liquid diets or promotility agents in minimizing aspiration risk.

## Figures and Tables

**Fig. (2):**
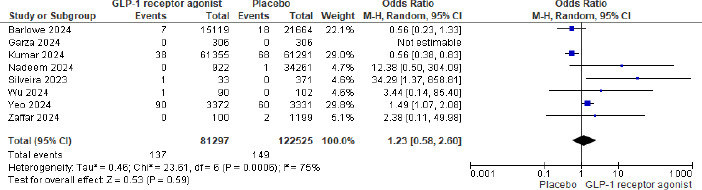
Forest plot and meta-analysis for pulmonary aspiration in patients on GLP-1 agonists and placebo.

**Fig. (3):**
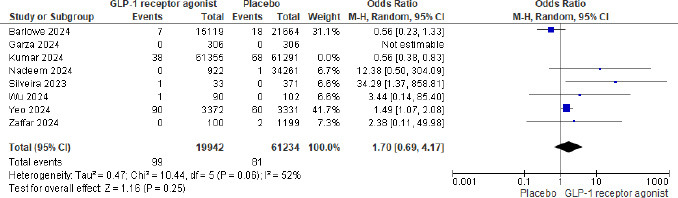
Forest plot for pulmonary aspiration in patients on GLP-1 agonists and placebo sensitivity analysis after excluding the study [27].

**Table 1:** The main characteristics of the included case reports and studies involving patients on GLP-1 agonists and placebo undergoing endoscopy.

**Author/Year/Refs**	**Type of Study**	**Country**	**Number of Patients**		**GLP-1 Agonist**	**Dose and Duration**	** GLP-1 agonist’s Hold Status and Duration **	**Fasting Duration**	**Indication for GLP-1 in Population**
Fujino 2023 [6]	Case Report	USA	1		Semaglutide	0.25mg once weekly	7 days before the procedure	10 hours	Type 2 DM, Obesity
Queiroz 2023 [17]	Case Report	Brazil	1		Semaglutide	0.5mg semaglutide SC	6 days before the procedure	9 hours	Obesity
Gulak 2023 [7]	Case report	Canada	1		Semaglutide	0.5mg SC once weekly	2 days before the procedure	20 hours for solids and 8 hours for clear fluids	Obesity
Klein 2023 [18]	Case report	USA	1		Semaglutide	1.7mg SC once weekly	18 hours	18 hours	Obesity
Avraham 2024 [5]	Case report	Italy	Case 1: 1 patient Case 2: 1 patient		Semaglutide	1mg SC once weekly	Case 1: 6 days prior Case 2: 4 days prior	Case 1: 12 hours Case 2: 8 hours	Type 2 DM
**Author/ year**	**Type of Study**	**Country**	**Number of patients** **On GLP-1 agonists**	**Number of patients** **on Placebo**	**GLP-1 agonist**	**Dose and duration**	** GLP-1 agonist’s Hold Status and duration **	**Fasting duration**	**Indication for GLP-1 in population**
Silveira 2023 [9]	Retrospective study	United States	1/33	0/371	Semaglutide	>30 days	11 days prior	12.4 hours	Obesity
Wu 2024 2023 [19]	Retrospective study	United States	1/90	0/102	Semaglutide 70, liraglutide 11, dulaglutide 6, tirzepatide 1, and a combination of two different drugs	329 (182–646) days	-	16 (14–19) hrs.	Type 2 DM, Obesity
Yeo 2024 [20]	Retrospective analysis of a large database	United States	90/3372	60/3331	-	-	-	-	Type 2 DM, Obesity
Anazco 2024 [21]	Retrospective analysis of a large database	United States	2/4134	-	Albiglutide, lixisenatide, exenatide, liraglutide, dulaglutide, semaglutide, tirzepatide	-	-	-	Type 2 DM, Obesity
Nadeem 2024 [22]	Retrospective study	United States	0/922	1/34261	-	-	-	-	Type 2 DM
Garza 2024 [23]	Retrospective case-control study	United States	0/306	0/306	-	-	-	At least 7 hours	Type 2 DM
Barlowe 2024 [24]	retrospective cohort study	United States	7/15119	18/21664	-	-	-	-	Type 2 DM
Maselli 2024 [25]	Retrospective study	United States	0/57	-	Semaglutide 30, liraglutide 11, dulaglutide 13, and tirzepatide 7	-	-	Solid foods 24 hours NPO 12 hours	Type 2 DM, Obesity
Firkins 2024 [26]	Retrospective study	United States	4/1897	-	Liraglutide 757, dulaglutide 396 and semaglutide 334	-	-	-	-
Kumar 2024 [27]	Retrospective cohort study	United States	38/61,355	68/ 61,291	-	-	-	-	-
Zaffar 2024 [28]	Retrospective study	Netherlands	0/100	2/1199	Semaglutide 41, and any GLP-1 agonist (semaglutide, dulaglutide, liraglutide, lixisenatide, exenatide, albiglutide, trizepatide)	-	-	-	-
Ghazanfar 2024 [29]	Retrospective cohort study	United States	0/306	-	-	-	-	126: clear liquid/low residue diet the day prior with NPO after midnight 180: Regular diet day prior with NPO after midnight	Type 2 DM

## Data Availability

The authors confirm that the data supporting the findings of this study are available within the article and its supplementary material.

## LIST OF ABBREVIATIONS

ORs: = Odds Ratios
POC: = Point of Care
RGC: = Residual Gastric Contents
GLP-1: = Glucagon-like Peptide-1

## STANDARD OF REPORTING

PRISMA guidelines and methodology were followed.

## SUPPLEMENTARY MATERIAL

PRISMA checklist is available as supplementary material on the publisher’s website along with the published article.
