# Supplementary material for: GLP-1 Agonists and the Risk of Pulmonary Aspiration during Elective Upper Endoscopy: A Systematic Review and Meta-analysis
Source: Open Respir Med J. 2025 Jun 11;19:e18743064372550. doi: 10.2174/0118743064372550250603061720 (PMC12481593; doi:10.2174/0118743064372550250603061720)
Supplement: Supplementary file 1 [file TORMJ-19-E18743064372550_SD1.pdf]

The Open Respiratory Medicine Journal

ISSN: 1874-3064

DOI: 10.2174/0118743064372550250603061720, 2025, 19, e18743064372550

SUPPLEMENTARY MATERIAL

OPEN ACCESS

GLP-1 Agonists and the Risk of Pulmonary Aspiration during Elective Upper Endoscopy: A Systematic Review and Meta-analysis

Praveen Reddy Elmati[1], Gowthami Sai Kogilathota Jagirdhar[2], Rakhtan K. Qasba[3], Andres Perez[4], Ruman K. Qasba[5], Yatinder Bains[2], Mehul Shah[2] and Salim Surani[6,*]

[1]*Department of Anesthesiology, Saint Clare's Hospital, Dover, NJ 07081, USA*
[2]*Department of Gastroenterology, Saint Michaels Medical Center, Newark, NJ 07104, USA*
[3]*Department of Medicine, Green Life Medical College and Hospital, Dhaka, Bangladesh*
[4]*Department of Medicine, Saint Francis Health Systems, Tulsa, NJ, USA*
[5]*Department of Medicine, Sher-i-Kashmir institute of medical sciences, Srinagar, Jammu and Kashmir, India*
[6]*Department of Medicine & Pharmacology, Texas A&M University, College Station, TX 77843, Texas, USA*

© 2025 The Author(s). Published by Bentham Open.
This is an open access article distributed under the terms of the Creative Commons Attribution 4.0 International Public License (CC-BY 4.0), a copy of which is available at: https://creativecommons.org/licenses/by/4.0/legalcode. This license permits unrestricted use, distribution, and reproduction in any medium, provided the original author and source are credited.

[*] Address correspondence to this author Department of Medicine & Pharmacology, Texas A&M University, College Station, TX 77843, Texas, USA; E-mail: srsurani@hotmail.com

Cite as: Elmati P, Jagirdhar G, Qasba R, Perez A, Qasba R, Bains Y, Shah M, Surani S. GLP-1 Agonists and the Risk of Pulmonary Aspiration during Elective Upper Endoscopy: A Systematic Review and Meta-analysis. Open Respir Med J, 2025; 19: e18743064372550. http://dx.doi.org/10.2174/0118743064372550250603061720

Received: December 26, 2024
Revised: April 12, 2025
Accepted: April 30, 2025
Published: June 11, 2025

Send Orders for Reprints to reprints@benthamscience.net

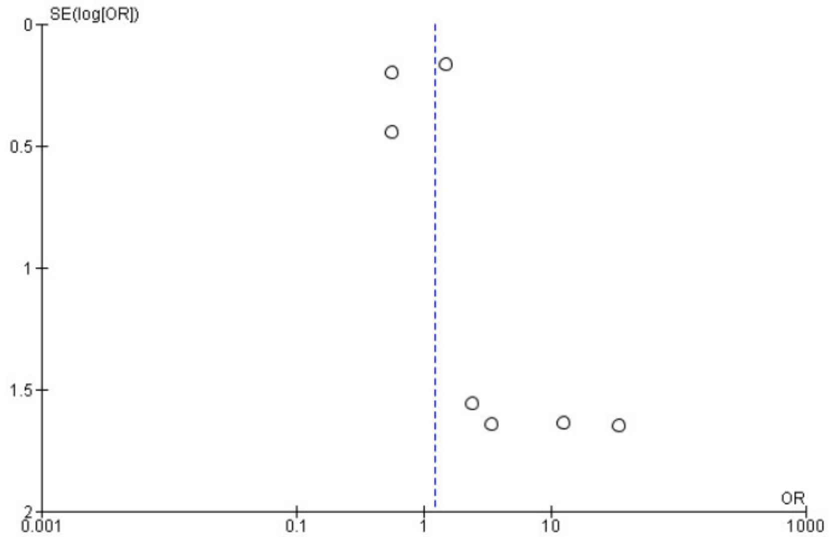

Fig. (S1). Publication Bias for Pulmonary aspiration in patients on GLP1 agonists and Placebo Meta-analysis *(A higher resolution / colour version of this figure is available in the electronic copy of the article).*

Table S1. Quality assessment of the studies based on the Joanna Briggs Institute (JBI) critical appraisal checklist for case reports.

Author	Were patient's demographic characteristics clearly described?	Was the patient's history clearly described and presented as a timeline?	Was the current clinical condition of the patient on presentation clearly described?	Were diagnostic tests or assessment methods and the results clearly described?	Were the definitions, inclusion and exclusion criteria, algorithms or processes used to identify or select cases and controls valid, reliable, and implemented consistently across all study participants	Was the intervention(s) or treatment procedure(s) clearly described?	Was the post-intervention clinical condition clearly described?	Was there use of concurrent controls	Were adverse events (harms) or unanticipated events identified and described?	Does the case report provide takeaway lessons?
**Fujino** [6]	√	√	√	√	√	√	√	√	√	√
**Gulak** [7]	√	√	√	√	√	√	√	√	√	√
**Klein [18]**	√	√	√	√	√	√	√	√	√	×
**Queiroz** [17]	√	√	×	√	√	√	√	√	√	√
**Avraham** [5]	√	√	√	√	√	√	√	√	√	√

Table S2. Quality assessment of the studies based on the National Institute of health quality appraisal tool for cohort and cross-sectional studies.

Author, years	Was the research question clearly stated	Was the study population clearly specified and defined	Was participation rate of eligible person at least 50%	Were all the subjects selected or recruited from the same or similar populations	Was a sample size justification, power description, or variance and effect estimates provided	Were the exposure(s) of interest measured prior to the outcome(s) being measured	Was the timeframe sufficient so that one could reasonably expect to see an association between exposure and outcome	Did the study examine different levels of the exposure as related to the outcome	Were the exposure measures clearly defined, valid, reliable, and implemented consistently across all study participants	Was the exposure(s) assessed more than once over time	Were the outcome measures clearly defined, valid, reliable, and implemented consistently across all study participants	Were the outcome assessors blinded to the exposure status of participants	Was loss to follow-up after baseline 20% or less	Were key potential confounding variables measured and adjusted statistically for their impact on the relationship between exposure(s) and outcome(s)
**Anazco, 2024** [21]	√	√	√	√	×	√	√	×	√	×	√	×	×	×
Barlowe 2024 [24]	√	√	√	√	√	√	√	×	√	×	√	×	×	√
Firkins 2024 [26]	√	√	√	√	×	√	√	×	√	×	√	×	√	×
**Gazabfar, 2024 [29]**	√	√	√	√	×	√	√	√	√	×	√	×	√	×
**Kumar, 2024** [27]	√	√	√	√	×	√	√	√	√	×	√	×	×	×
**Maselli, 2024** [25]	√	√	√	√	√	√	√	√	√	×	√	×	×	×
**Nadeem, 2024** [22]	√	√	√	√	×	√	√	√	√	×	√	×	×	√
**Silveira, 2023** [9]	√	√	√	√	√	×	√	√	√	×	√	×	×	×
**Wu, 2024 [19]**	√	√	√	√	√	×	√	√	√	×	√	×	×	×
**Yeo 2024 [20]**	√	√	√	√	√	×	√	√	√	×	√	×	×	×
**Zaffar, 2024** [28]	√	√	√	×	×	√	√	√	√	×	√	×	×	√

Table S3. Quality assessment of the studies based on the National Institute of health quality appraisal tool for Case control studies.

Author, years	Was the research question or objective in this paper clearly stated and appropriate	Was the study population clearly specified and defined	Did the authors include a sample size justification	Were controls selected or recruited from the same or similar population that gave rise to the cases (including the same timeframe)	Were the definitions, inclusion and exclusion criteria, algorithms or processes used to identify or select cases and controls valid, reliable, and implemented consistently across all study participants	Were the cases clearly defined and differentiated from controls	If less than 100 percent of eligible cases and/or controls were selected for the study, were the cases and/or controls randomly selected from those eligible	Was there use of concurrent controls	Were the investigators able to confirm that the exposure/risk occurred prior to the development of the condition or event that defined a participant as a case	Were the measures of exposure/risk clearly defined, valid, reliable, and implemented consistently across all study participants	Were the assessors of exposure/risk blinded to the case or control status of participants	Were key potential confounding variables measured and adjusted statistically in the analyses, If matching was used, did the investigators account for matching during study analysis
**Garza ,2024** [23]	√	√	×	√	√	√	×	×	×	√	×	×
